# Experimental and Numerical Study on Various Liquid-Cooled Heat Sinks Including Topology Optimization

**DOI:** 10.3390/mi16111213

**Published:** 2025-10-25

**Authors:** Ge Shi, Yanwei Dai, Zhongjun Yu, Xingyao Zeng, Cheng Tan, Haibo Zhang, Guang Yang, Fei Qin

**Affiliations:** 1 Institute of Electronics Packaging Technology and Reliability, School of Mathematics, Statistics and Mechanics, Beijing University of Technology, Beijing 100124, China; shigegump@163.com (G.S.); yg15201312732@163.com (G.Y.); qfei@bjut.edu.cn (F.Q.); 2 Aerospace Information Research Institute, Chinese Academy of Sciences, Beijing 100094, China; cengxy@aircas.ac.cn (X.Z.); tancheng@aircas.ac.cn (C.T.); zhanghb000046@aircas.ac.cn (H.Z.)

**Keywords:** liquid-cooled, topology optimization, millimeter-wave antenna, heat dissipation, pressure drop

## Abstract

With the rapid advancement in millimeter-wave antennas, heat dissipation of array heat sources faces significant challenges due to increasing requirements on power density and miniaturization. Efficient thermal management is essential to ensure reliability and performance, particularly in high-power applications such as millimeter-wave antennas. This study investigates liquid-cooled heat sinks featuring an inlet and outlet on the same side. A topology optimization method is employed to design an efficient heat sink, minimizing both average temperature and fluid dissipation. The optimized design is compared with common liquid-cooled heat sinks, including series, parallel, pin rib, and tree channels, through experimental and numerical simulations. A dedicated liquid-cooled experimental platform is developed to evaluate thermal and fluid characteristics under different flow rates. The results demonstrate that the topology optimization channels achieve superior thermal uniformity and lower pressure drop compared to traditional designs. The average temperatures of the heat sources in topology optimization I and II channels are 6% and 4% lower than those in the other flow channels, respectively, and the topology optimization I channel exhibits the most favorable fluid characteristics, with a pressure drop 9% lower than that of the parallel flow channel. Specifically, the topology optimization I and II channels exhibit balanced heat dissipation and flow resistance, while the series channel suffers from excessive pressure losses. The findings provide valuable insights for optimization, offering a practical method for enhancing thermal management in millimeter-wave antenna applications.

## 1. Introduction

The rapid advancement in millimeter-wave antenna technologies has triggered significant thermal challenges, as elevated temperatures adversely affect the reliability and performance of electronic components [1]. Studies indicate that overheating is a primary cause of electronic device failure, with improper thermal management leading to degraded system performance, reduced efficiency, shortened life, and even catastrophic failures [2,3]. A plurality of factors impact the thermal behavior, including heat from adjacent chips, airflow, packaging materials, fabrication processes, lead wires, and ambient temperature [4]. Excessive junction temperatures in microwave integrated circuits (MMICs) can severely compromise reliability, potentially resulting in device failure or burnout [5].

In aerospace and automotive applications, the widespread adoption of millimeter-wave antennas is aimed toward higher integration and performance [6]. This progress is accompanied by challenges, particularly in thermal management. Recent studies reveal that the heat flux density of a power amplifier chip (PA chip) has exceeded 100 W/cm^2^ and is aimed to reach 1000 W/cm^2^ [7]. Efficient heat dissipation under such extreme conditions is critical, as temperature rise directly impacts device reliability. Addressing thermal issues is, therefore, essential to prevent performance drift and thermal failure in millimeter-wave antennas.

Traditional cooling methods include active and passive techniques such as air cooling, liquid-cooled [8,9,10,11,12], phase-change material cooling [13,14,15,16], and heat pipe cooling [17]. Air cooling is still extensively utilized owing to its cost-effective operation and simple maintenance. Nevertheless, low thermal conductivity and specific heat limit its applicability in high-heat-flux scenarios [17,18]. Heat pipes leverage phase-change mechanisms for rapid heat transfer, outperforming all known metals in thermal conductivity, albeit at higher costs [19]. Phase-change materials exploit latent heat absorption to achieve excellent temperature uniformity at low flow rates but are less effective for continuously dissipating components [20]. Liquid-cooled, which relies on forced convection between a coolant and solid walls, offers high heat transfer efficiency, sustained cooling capacity, and cost-effective manufacturability (e.g., metal heat sink [20]), making it a promising solution for high-heat-flux applications.

Designing high-performance liquid-cooled heat sinks remains a complex task, often requiring iterative prototyping based on experimental validation. This trial-and-error approach prolongs development cycles and increases costs. Shen et al. [21] employed numerical simulations to optimize thermal performance of multi-channel liquid-cooled battery, and identified the optimal flow rates. Husain et al. [22] explored fluid flow and heat transfer in rectangular microchannels, while Peles et al. [23] investigated the thermal–fluid performance of pin rib air-cooled heat sinks. Topology optimization has since revolutionized liquid-cooled plate design. Since Michel’s pioneering work on truss optimization in 1904 [24], building on Maxwell’s stress constraints [25], various topology optimization methods have emerged, including the relative density method, level-set method [26], and homogenization method [27]. In thermal management, Srinivas et al. [28] demonstrated a 35% enhancement in heat transfer using SIMP (Solid Isotropic Material with Penalization) optimized aluminum heat sinks with phase-change materials. Baja et al. [29] improved microchannel flow distribution via surface topology modifications, and Li et al. [30] validated the superiority of a 2D topology-optimized heat sink over traditional designs. Concurrently, the application of density-based topology optimization under turbulent conditions and its utilization in optimizing needle-fin arrays have garnered significant scholarly attention. Topology optimization of turbulent fields has also played a pivotal role in enhancing cooling applications [31,32,33].

Despite extensive research on liquid-cooled systems, studies on heat sinks with inlets and outlets on one side remain scarce. Variability in sink dimensions, heat source, and experimental conditions complicates result comparability. Key challenges include optimizing cooling performance under constrained boundary conditions, minimizing pressure drop, and ensuring operational stability.

**Figure 1:**
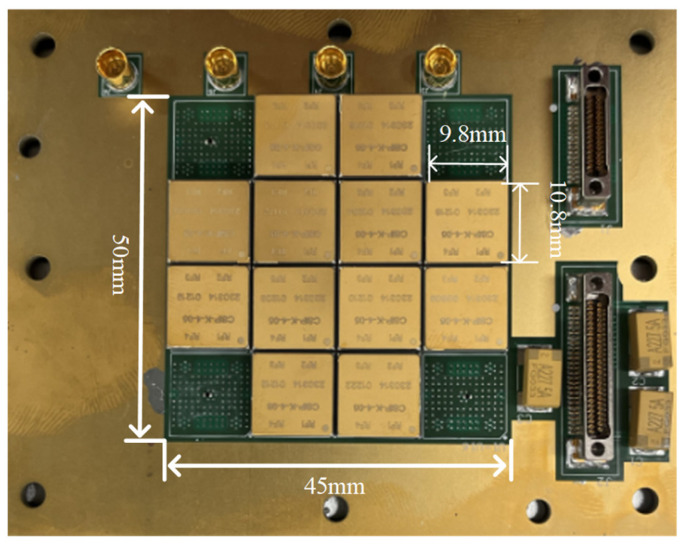
The front of the T/R component.

**Figure 2:**
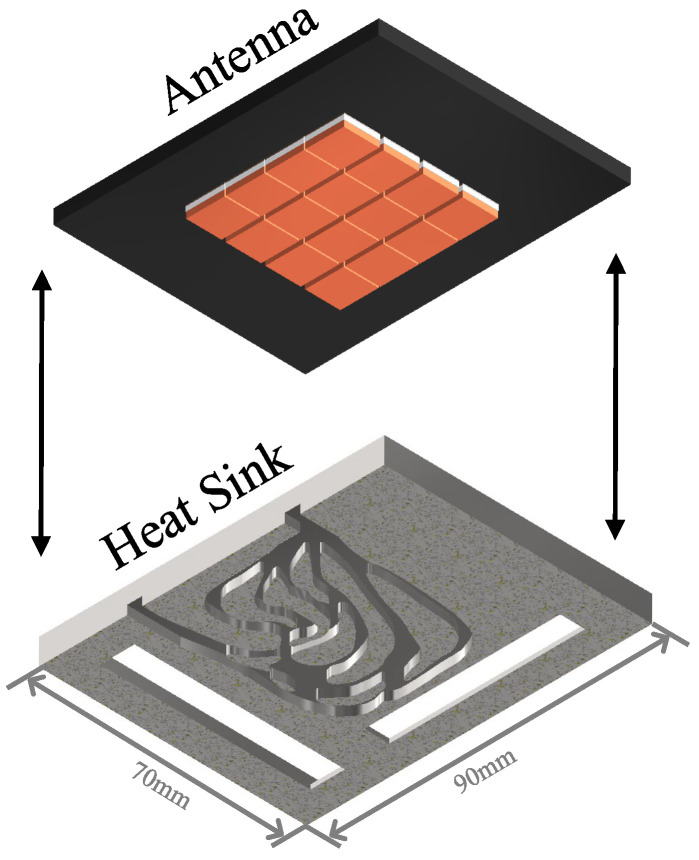
Assembly model of liquid-cooled heat sink.

To address the thermal management challenges of a millimeter-wave antenna with sixteen transmitter and receiver (T/R) components (Figure 1), this study designs with an inlet and outlet on one side (Figure 2) for high heat flux applications. The heat flux originates from four amplifier chips with 0.4 W mounted on a 100 mm^2^ substrate.

We propose flow channel designs, including the series [34,35], parallel [36], and pin rib heat sinks, alongside a topology optimization sink derived from COMSOL Multiphysics 6.2, which minimizes average temperature and flow dissipation energy via weighted multi-objective optimization. Thermal analysis and pressure drop analysis of the flow path were conducted using Ansys Icepak 2021, with the solver retrieved from Fluent. Heat sinks were machined using CNC (Computer Numerical Control) with aluminum and evaluated on a liquid-cooled experimental platform to compare thermal and flow characteristics.

## 2. Topology Optimization Design of Heat Sink

Topology optimization serves as a mathematical tool, optimizing the material distribution within a given design area based on the given load conditions (thermal load, force load, etc.), constraints, and performance indicators. This method can combine the optimization theory in mathematics with engineering design, so that the final scheme can meet the engineering requirements, and achieve the best performance compared with other schemes, so as to improve the engineering efficiency and design quality.

### 2.1. Topology Optimization Model

**Figure 3:**
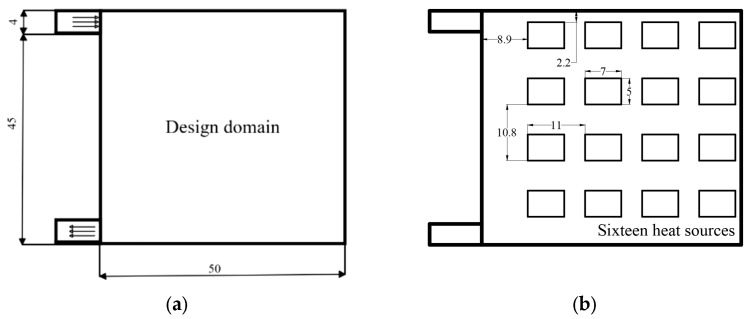
(**a**) Topology optimization design domain of liquid-cooled heat sink; (**b**) the heat flux density distribution, dimensions, and relative positions of the heat sources.

In order to save time and calculation cost, the two-dimensional fluid topology optimization calculation is carried out for the cross-sectional profile of the liquid-cooled unit with an inlet and outlet on the same side. The calculation domain includes the topology optimization calculation area and the two-dimensional fluid inlet and outlet area, where the topology optimization calculation area is a 45 mm × 50 mm solid/fluid heat transfer physical field calculation area. The fluid inlet and outlet are defined as rectangular fluid physical field calculation areas with a width of 4 mm ×8 mm, where the arrow direction is the direction of fluid flow. The design domain, the heat flux density distribution of the heat sources, and their dimensional parameters are shown in Figure 3.

### 2.2. Computational Methods for Topology Optimization

Topology optimization methods evolved from structural optimization techniques and have seen expanded application in fluid heat transfer in recent years. Addressing the physical field of fluid flow, Borrvall and Petersson [37] pioneered the introduction of a Brinkmann [38] penalty term into the momentum equation, thereby enabling topology optimization within Navier–Stokes flows. Subsequently, the topo method was employed to investigate minimizing dissipated power within fluids. For thermal conduction problems, topological structures enable the optimal distribution of highly thermally conductive materials, directing heat transfer towards idealized ‘cold spots’ [39]. This has been extensively investigated through various topological optimization methods, such as density-based algorithms [40]. After addressing thermal resistance problems concerning fluid flow and heat conduction separately, a multi-objective function is typically formulated using two linearly weighted criteria: one for fluid flow and another for thermal considerations. This approach aims to maximize heat transfer efficiency within the design domain while maintaining a low pressure drop. According to this theoretical methodology, Okkels investigated fluid–thermal topology optimization problems [41].

#### 2.2.1. Boundary Conditions

For fluid fields, the Navier–Stokes equation with boundary conditions is given by Equation (1)

(1) $$\rho \left(\frac{\partial u}{\partial t}+u\cdot \nabla u\right)=-\nabla p+\mu \nabla^{2}u+f$$

In topology optimization, the fluid is considered as a two-dimensional steady model viscous unconstrained fluid, and a momentum equation with a damping term is added to calculate the fluid resistance in the porous media of the model fluid, and the expression is shown in Equation (2) [42].

(2) $$\rho \left(\frac{\partial u}{\partial t}+u\cdot \nabla u\right)=-\nabla p+\mu \nabla^{2}u-\alpha u$$

where α denotes the damping coefficient, whose physical significance is the reverse permeability of the porous medium. By setting the value of α, exchange between the fluid phase and the solid phase can be achieved; the specific mechanism will be detailed in the subsequent sections.

Heat sink is achieved by heat conduction and heat convection. The fluid flow in the topologically optimized region is a laminar flow model, and the physical field of heat transfer between the solid and fluid. In order to simulate the heat source of the T/R component, sixteen heat sources were set in the design domain to replace the heat consumption of the T/R component. The width of the fluid inlet was 4 mm, and the flow rate was 0.1 m/s. The flow rate corresponding to this flow rate can be calculated by the laminar flow model. The boundary of the design domain was set to be thermally insulated.

#### 2.2.2. Calculation of Material Interpolation

Material interpolation calculation for topology optimization is intended to identify the optimal distribution of materials in the design domain, among which the variable density method is a topology optimization method widely used in heat sink design [43,44]. In this method, the material density within the design domain varies continuously, and it can gradually transition from solid to fluid, forming an intermediate state. The relative density and material properties are linked by the interpolation function model, which interpolates the various physical characteristics of the liquid-cooled plate material between the fluid and the solid. RAMP (Rational Approximation of Material Properties) is employed to interpolate the reverse osmosis coefficient. Equation (3) illustrates the method of expression.

(3) $$\alpha =\alpha_{\min}+(\alpha_{\max}-\alpha_{\min})\frac{q(1-\gamma )}{q+\gamma }$$

The material phase in the design problem is regarded as a porous medium, and the cell material density is represented by the design variable γ (0 < γ < 1). When γ = 0, the cell is filled with fluid, and when γ = 1, the cell is filled with solid. αmin and αmax denote the minimum and maximum values of α, which correspond to the reverse osmotic pressure of the fluid and solid, respectively. In addition, q is the penalty parameter adopted to regulate the shape of the function α. The calculation formula is shown in Equation (4). Da is the Darcy number and describes the relationship between viscous force and friction in porous media. μ is the dynamic viscosity of the fluid, and L is the characteristic length of the inlet section.

(4) $$\alpha_{\max}=\frac{\mu }{\mathrm{Da}\cdot L^{2}}$$

Physical properties are interpolated using the SIMP method [45], and Equation (5) demonstrates the calculation approach for the reverse osmosis coefficient. k represents the thermal conductivity, k_s_ represents the solid thermal conductivity, and k_f_ represents the liquid thermal conductivity.

(5) $$k=k_{s}+\gamma (k_{f}-k_{s})$$

In topology optimization calculation, the specific heat C_p_ calculation formula is shown in Equation (6), where the specific heat of solids is C_ps_ and the specific heat of liquids is C_pf_.

(6) $$C_{p}=C_{\mathrm{ps}}+\gamma (C_{\mathrm{pf}}-C_{\mathrm{ps}})$$

#### 2.2.3. Density Filtering and Projection

Density filtering and projection techniques are typically employed to prevent numerical instability in the topology optimization process, such as mosaic and grid dependency [45]. In order to accomplish density filtering, Kawamoto et al. [46] suggested a Heaviside projection technique based on the Helmholtz partial differential equation. The procedure is displayed in Equation (7). The projection parameter β, which is managed by a smoothing function that can map density values between 0 and 1, determines the physical density that is obtained through projection. Filter density values over the threshold γ_β_ are projected as 1 during projection, whereas those below the threshold γ_β_ are projected as 0.

(7) $$\gamma =\frac{\tanh(\beta (\gamma_{f}-\gamma_{\beta }))+\tanh(\beta \gamma_{\beta })}{\tanh(\beta (1-\gamma_{\beta }))+\tanh(\beta \gamma_{\beta })}$$

#### 2.2.4. Topology Optimization Objective

Within the field of topology optimization, the objective associated with optimization simulation consists of two key aspects: the minimization of fluid dissipated energy and the minimization of average temperature. In the domain, the dissipation energy of the fluid represents the amount of kinetic energy dissipated as the fluid moves. Minimizing the dissipation energy can make the fluid flow at a higher velocity in the flow channel and take away more heat. When the average temperature reaches its minimum value, the heat source’s overall temperature declines, and the heat source’s temperature becomes relatively consistent.

The optimization equation is expressed by the ratio of the optimized variable ($\mathrm{ob}j_{i}$) to the corresponding variable ($\mathrm{ob}j_{t}$/$\mathrm{ob}j_{u}$), without topology optimization, as shown in Equations (8) and (9), so that the initial value of both optimization objectives is set to 1 before optimization starts. Ω is the design domain. $\mathrm{ob}j_{t}\;\mathrm{and}\;\mathrm{ob}j_{u}$, respectively, represent minimization objective of the average temperature and minimization of total pressure drop. α(γ) represents inverse permeability. u is the velocity vector.

(8) $$\mathrm{ob}j_{1}=\int_{\Omega }\left[\frac{1}{2}\mu (\nabla u+\nabla u^{T}):(\nabla u+\nabla u^{T})+\alpha (\gamma )(u\cdot u)\right]d\Omega /\mathrm{ob}j_{u}$$

(9) $$\mathrm{ob}j_{2}=\left(\int T\;d\Omega /\Omega \right)/\mathrm{ob}j_{t}$$

The final topology optimization objective J is represented by the weighted sum of the two normalized optimization objectives, and the sum of the weight factors is 1, as shown in Equation (10). This weighting method, w_1_ + w_2_ = 1, provides a flexible optimization strategy to achieve a balance between minimizing fluid dissipation energy and minimizing average temperature to meet different design requirements.

(10) $$J=w_{1}\mathrm{ob}j_{1}+w_{2}\mathrm{ob}j_{2}$$

We selected the SIMP method for interpolating material properties while simultaneously imposing material volume constraints. Consequently, the flow channel layout topology optimization model can be formulated as follows:

(11) $$\begin{array}{cc} find & \gamma \\ \min & J=w_{1}{\mathrm{obj}}_{1}+w_{2}{obj}_{2}\\ s.t. & \nabla \cdot u=0\\ & \rho (u\cdot \nabla )u=-\nabla p+\mu \nabla^{2}u-\alpha u\\ & \alpha (\gamma )=\alpha_{min}+(\alpha_{max}-\alpha_{min})\frac{q(1-\gamma )}{q+\gamma }\\ & \rho C_{p}(u\cdot \nabla )T=k\nabla^{2}T+(1-\gamma )\theta (T_{Q}-T)\\ & k=k_{s}+\gamma^{p}(k_{s}-k_{f})\\ & \frac{1}{V}\int_{\Omega }\gamma \;d\Omega -V^{*}<0\\ & 0\leq \gamma \leq 1 \end{array}$$

### 2.3. Topology Optimization Results

**Figure 4:**
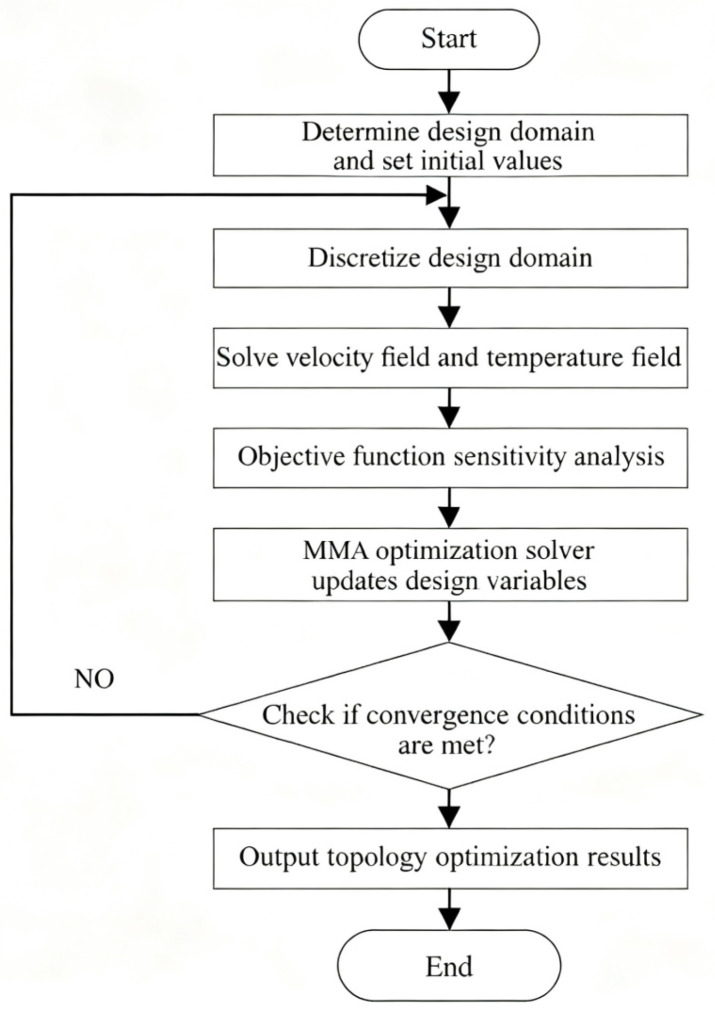
Flow path topology optimization flowchart.

The aforementioned issues were implemented in this paper using the finite element software COMSOL Multiphysics 6.2. The flow field analysis employed the software’s CFD module to solve the second-order discretization of velocity and the first-order discretization of pressure. The heat transfer process analysis utilized the software’s heat transfer module to solve the convective diffusion equation and the thermal diffusion problem within the substrate. The parallel sparse direct solver PARDISO provided by COMSOL was employed to solve the discretized finite element equations, whilst separate solution steps addressed the fluid, heat transfer, and optimization problems. As the velocity, pressure, and temperature fields, along with the objective function, implicitly depend on the design variable γ, the derivatives of the objective function with respect to the design variable could not be computed directly. Consequently, the adjoint variable method was adopted for the sensitivity analysis. The flow channel topology optimization workflow is illustrated in Figure 4.

The topology optimization solution employed the Method of Moving Asymptotes (MMA) as its iterative optimization algorithm. MMA excels at handling diverse multiphysics topology optimization problems and has gained widespread application within the field. This algorithm is integrated within the software package, with the optimization iteration ceasing only upon fulfillment of the convergence criteria. The convergence criterion is set as follows: $\varepsilon =\left|J^{k+1}-J^{k}\right|\leq 10^{-5}$. Additionally, to mitigate grey cells and mesh dependency in the topology optimization, the methodology outlined in the preceding section was employed: density filtering was achieved using a Helmholtz partial differential equation filter, while hyperbolic tangent projection was applied to minimize grey cell generation, thereby yielding a clear flow channel topology.

By referencing other researchers’ work on topology optimization parameters [30] and following analysis and validation through simulation, we selected the topology optimization parameters, particularly confirming those related to reverse permeability, as shown in Table 1. Based on this table and Equation (3), the parameter α_max_ can be determined. And α_min_ was set to a negligibly small value to ensure that fluid flow was effectively blocked in solid regions (where γ = 0). And this paper set the initial value of volume fraction V_0_ to 0.5.

**Figure 5:**
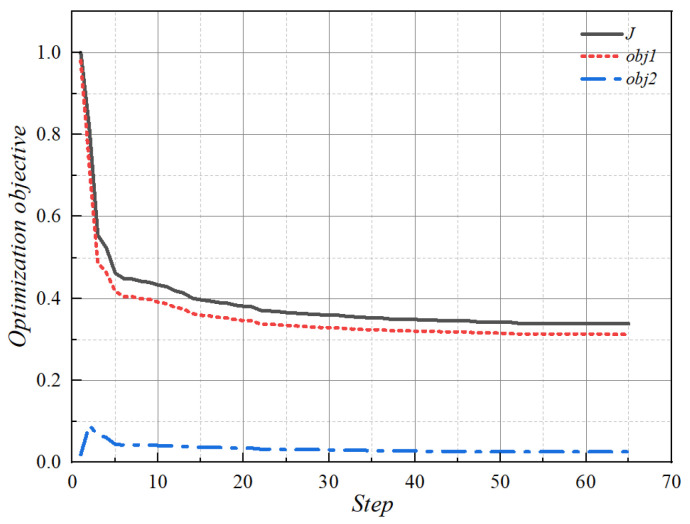
Results of topology optimization objective of liquid-cooled heat sink with the number of iteration steps.

According to the above principles and algorithms, the topology optimization of two optimization objects with different w_1_ and w_2_ values was calculated in COMSOL Multiphysics software, and the proportion of the restricted fluid region in the optimization process was 0.5. The machinability of the result should be considered in order to obtain the topology optimization result of the liquid-cooled heat sink in the calculation process. The influence of weight factors w_1_ and w_2_ on the results is that when the temperature weight is large, the topology optimization results have more fluid branches and uniform coverage. When the fluid characteristic weight is large, the fluid branches are few and wide, and the fluid coverage is not comprehensive. After the final calculation, it is easy to process, and the final optimization target value is minimum. Figure 5 demonstrates the correlation between the target result and the number of iterations. At the initial stage of optimization, the mean temperature of the heat source is reduced, and fluid dissipation grows substantially. As iterations go on, both the heat source’s mean temperature and fluid dissipation decrease.

**Figure 6:**
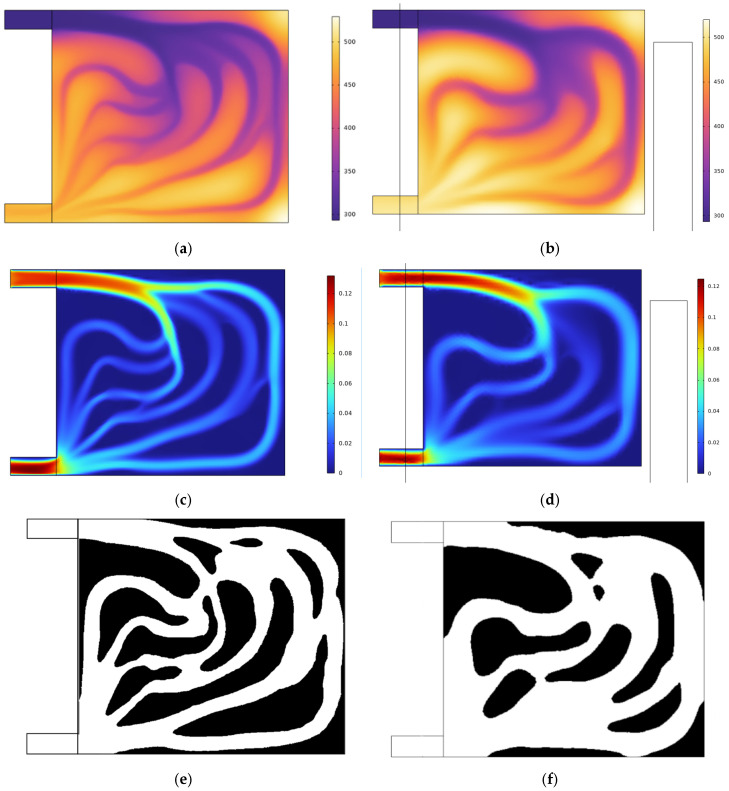
Flow channel structure of liquid-cooled topology optimization results: (**a**) temperature result with the volume fraction 0.5; (**b**) temperature result with the volume fraction 0.7; (**c**) flow rate result with the volume fraction 0.5; (**d**) flow rate result with the volume fraction 0.7; (**e**) topology optimization results with the volume fraction 0.5; (**f**) topology optimization results with the volume fraction 0.7.

The flow channel structure obtained by topology optimization is demonstrated in Figure 6. By obtaining the topology optimization result when the projection threshold value γ_β_ is greater than 0.5, it is the fluid region [30], that is, the volume fraction 0.5 (as shown in Figure 6a), and the rest is the solid region. By implementing the above settings and performing the calculations, a two-dimensional cross-section diagram of the liquid-cooled plate can be obtained, in which the double objective weight factor w_1_ = 0.9 and w_2_ = 0.1 in Equation (8). Finally, for the purpose of comparing the optimization results across different proportions, the topology-optimized structure of the liquid-cooled heat sink was obtained by limiting the volume fraction ratio to 0.7, as illustrated in Figure 6b. It can be known that topology optimization I, series, parallel I, circular pin rib, triangular pin rib, parallel II, tree, and topology optimization II channel structure all employ a volume ratio of 0.5. This uniform volume fraction of 0.5 across all the channels facilitates a fair longitudinal comparison. The volume fraction 0.7 is used to enable a lateral comparison with the topology optimization results achieved at the 0.5 volume ratio.

**Figure 7:**
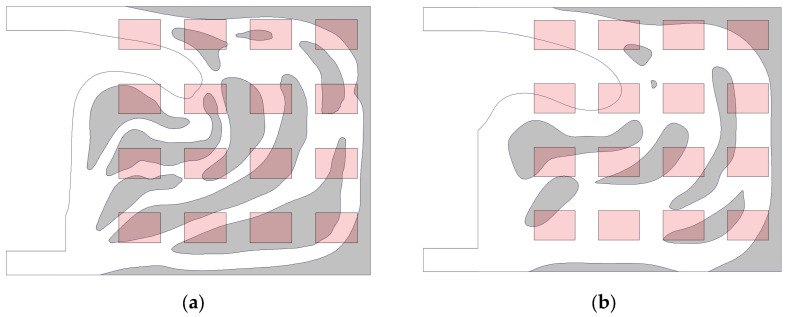
Heat flux density distribution (pink) and flow channel topology optimization results (light grey): (**a**) the volume fraction 0.5; (**b**) the volume fraction 0.7.

As illustrated in Figure 7, the pink regions denote array heat sources, whose spatial distribution determines the thermal flux density distribution.

The central region within the design domain exhibits a concentration of heat sources, resulting in locally elevated thermal flux densities. These areas necessitate enhanced heat dissipation measures to prevent overheating. It is evident that both volumetric fraction flow channels feature adaptive, densely branched channels in regions of higher thermal flux density. This ensures sufficient coolant flow to dissipate the concentrated heat generation.

More sparsely distributed heat sources, such as those at the periphery of the design domain, correspond to relatively lower heat flux densities. The flow channels at the domain edges adopt streamlined configurations. This approach avoids redundant flow paths, thereby preventing increased pressure drop along the flow path, while maintaining fundamental heat transfer efficacy.

Physically, this can be understood as liquid-cooled structures being custom-designed through topology optimization methods. The shape and orientation of the flow channels are determined by the heat flux density imposed by the heat source array. The response is achieved by allocating channel morphology according to the heat flux density distribution. This approach generates solutions that balance thermal performance while satisfying given volume constraints, whether under volume fraction constraints of 0.5 or 0.7. The topology optimization algorithm links the heat source distribution, heat flux density, and channel topology.

## 3. Numerical Investigation of Liquid-Cooled Heat Sink

This study explores the heat dissipation performance and fluid characteristics of the topology-optimized liquid-cooled heat sink, and compares the heat dissipation performance of the topology-optimized channel to the common flow channel. Suggestions on how to select a suitable liquid-cooled heat sink structure with a heat source array are given. The numerical results in this section are based on the model validated by experimental data in Section 4.2, for details, with key indicators showing <10% error.

### 3.1. Numerical Modeling and Boundary Conditions

**Figure 8:**
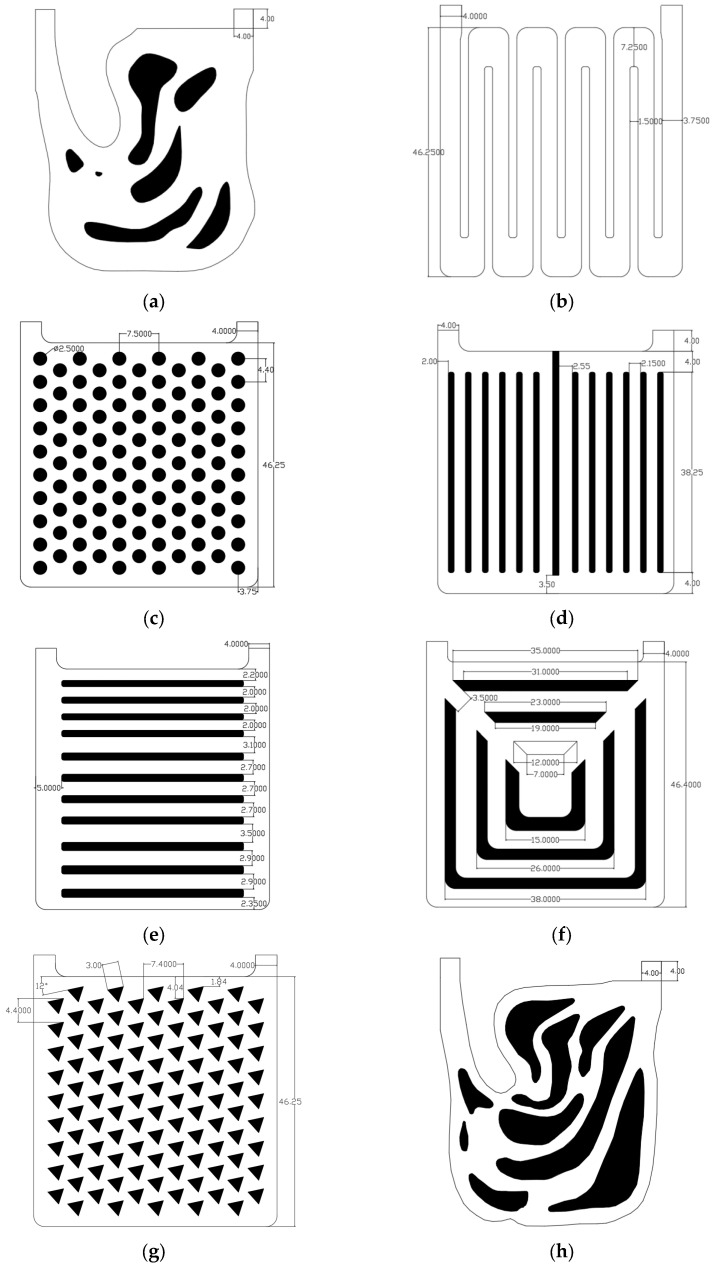
Liquid-cooled channel designs in heat sinks: (**a**) topology optimization I channel; (**b**) series channel; (**c**) circular pin rib channel; (**d**) parallel I channel; (**e**) parallel II channel; (**f**) triangular pin rib channel; (**g**) tree channel; (**h**) topology optimization II channel.

In order to examine the heat dissipation capability and fluid characteristics of different liquid-cooled heat sinks, including topology optimization, flow channel heat sinks with a fluid proportion of 0.7 and 0.5 were designed. Those proportions were balanced with manufacturability and heat exchange area, especially when the proportion of topology optimization results was 0.5. The designed flow channel forms in heat sinks mainly included the following: two parallel channels as illustrated in Figure 8d,e, a series channel as illustrated in Figure 8b, a pin rib channel as illustrated in Figure 8c,g, a tree channel [47] as illustrated in Figure 8g, and topology optimization channels as illustrated in Figure 8a,h. Parallel channel I was designed with the ribs and the inlet/outlet parallel to each other, and Parallel channel II was designed vertically. The series type is an S-type flow channel without branches. The pin rib channels are flowing channel structures with triangular and circular rib columns evenly distributed in the flow channel. A tree channel heat sink has multiple branches covering the liquid-cooled area in the main channel. The black area in Figure 8 is the solid area, and the rest is the fluid.

Since there are many flow channel types, the flow channel names were simplified. The corresponding sequence numbers and simplified names are shown in Figure 8.

In the numerical simulation of fluid/solid coupled heat transfer boundary conditions, the ambient temperature, the boundary temperature of the computational domain, and the ambient thermal radiation temperature were all set to 20 °C. The calculation domain was a hexahedral space. The top and bottom were set as openings to simulate the actual work and external heat transfer.

The liquid-cooled heat sink inlet was selected to have an inlet flow rate of 80 mL/min (inlet flow rate of 0.1 m/s). The inlet flow temperature was 20 °C. The power of the MCH heat source was 1.6 W, and the overall power of the 16 heat sources was 25.6 W. The thermal–fluid coupling of the above liquid-cooled heat sinks was analyzed. The computational simulation model in this research was the same as the experimental model, and the main parameters of its material are represented in Table 2.

Figure 8 shows the structure of the liquid-cooled heat sink after the heat source has been assembled. In order to facilitate follow-up experimental research, sixteen Metal Ceramic Heaters (MCHs) were used to simulate T/R components. We fixed the MCH to the heat sink using a carrying plate, and used a thermal pad to fill the gap between the MCH and the heat sink.

**Figure 9:**
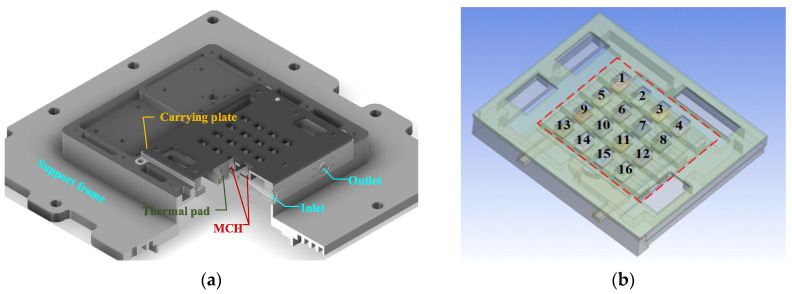
(**a**) The structure of the liquid-cooled heat sink; (**b**) MCH number and location.

Combining the parameters (such as thermal conductivity, specific heat capacity, etc.) and boundary conditions (such as heat source power, convective heat transfer coefficient, etc.) of the above materials, the heat-fluid coupling problem was analyzed in detail using the Ansys ICEPAK 2021 numerical simulation software. The numerical simulation model needed to be reasonably simplified, and its structure and heat source number are illustrated in Figure 9.

### 3.2. Mesh Sensitivity Analysis

Meshing serves as a key procedure in finite element numerical simulation, and its mesh quality directly affects the accuracy, stability, and efficiency of the solution. For the thermal–fluid coupling problem, reasonable mesh delineation needs to take into account the characteristics of multi-physical fields, and adopt the strategy of local encryption and adaptive adjustment of the boundary layer to attain the optimal balance between computational accuracy and cost.

Prior to carrying out the simulation, the mesh dependency of the numerical models was tested. The quantity of tetrahedral elements rose until both of the following criteria were met: ≤2.0%, with m denoting the index for the mesh sensitivity tes. The article selected the temperature of the topo I heat sink at a flow rate of 0.1 m/s (80 mL/min) for comparison. Table 3 presents the relative errors of peak temperature associated with the number of elements.

**Figure 10:**
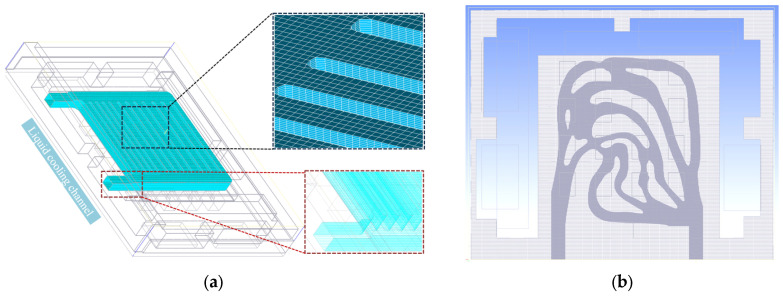
Numerical simulation meshing of liquid-cooled heat sink: (**a**) series channel; (**b**) topology optimization I channel.

It is evident that the numerical solutions achieve mesh independence once the element count reaches 6,568,243. The mesh quality directly determines the convergence performance and is an important prerequisite for verifying the credibility of the numerical simulation. In this study, the global mesh size was set to have a maximum mesh size of 0.4 mm and a minimum mesh size of 0.05 mm in the xy direction, and a maximum mesh size of 0.1 mm and a minimum mesh size of 0.01 mm in the z direction to ensure the balance between computational accuracy and efficiency. In order to more accurately capture the flow and heat transfer characteristics within the liquid-cooled flow channel, the computational region adopted a local encryption mesh method, and the local encryption region was the fluid boundary region. The maximum mesh size in the x, y direction mentioned above was adjusted to the same minimum mesh size of 0.2 mm, and the mesh size in the z direction remained unchanged. The mesh quality is shown in Figure 10, demonstrating the distribution and refinement of the mesh to ensure sufficient resolution in critical areas to support high-precision numerical simulations.

### 3.3. Simulation Results

#### 3.3.1. Simulation Results of Heat Dissipation Performance

**Figure 11:**
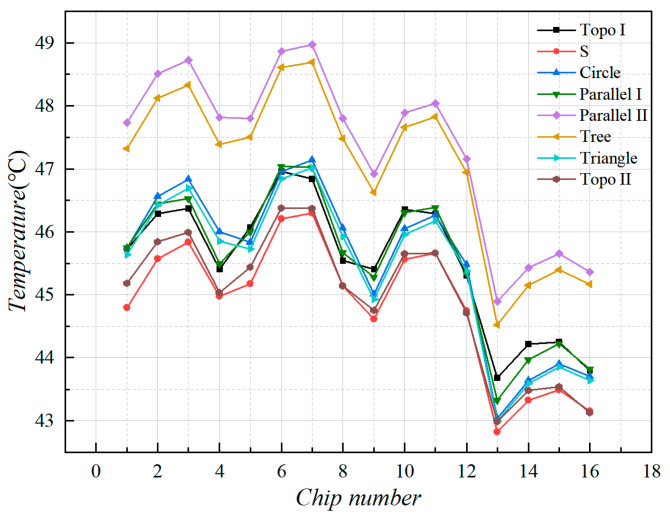
Temperature of each heat source in steady liquid-cooled environment at 80 mL/min inlet flow rate.

The maximum MCH temperature distribution under different liquid-cooled heat sinks is represented in Figure 11. As can be seen, the trend of MCH temperature distribution is basically the same for all the heat sinks. The simulation findings illustrate that under a 20 °C environment, 20 °C cooling medium at an 80 mL/min inlet flow rate, the heat source temperature of the S channel was the lowest, and the highest temperature rise in MCH was only 26.3 °C. The MCH temperature of the parallel II channel and the tree channel was higher, close to 29 °C. The MCH temperature of the topology-optimized II channel was almost the same as the heat source temperature of the S channel, with the highest temperature rise of 26.2 °C. The highest temperature of the remaining runner heat sink MCH was around 27 °C.

For the same runner heat sink, the difference between the maximum and minimum MCH temperatures was approximately 4.1 °C. By analyzing the temperatures corresponding to the MCH positions, it was found that the temperature of the heat sources at the corners of the MCH array was relatively low, followed by the heat sources at the edges of the array. And the chip in the middle area of the array had the highest temperature. The reason for this result was mainly due to the concentration of heat sources in the center part of the MCH array and the high density of heat flow.

**Figure 12:**
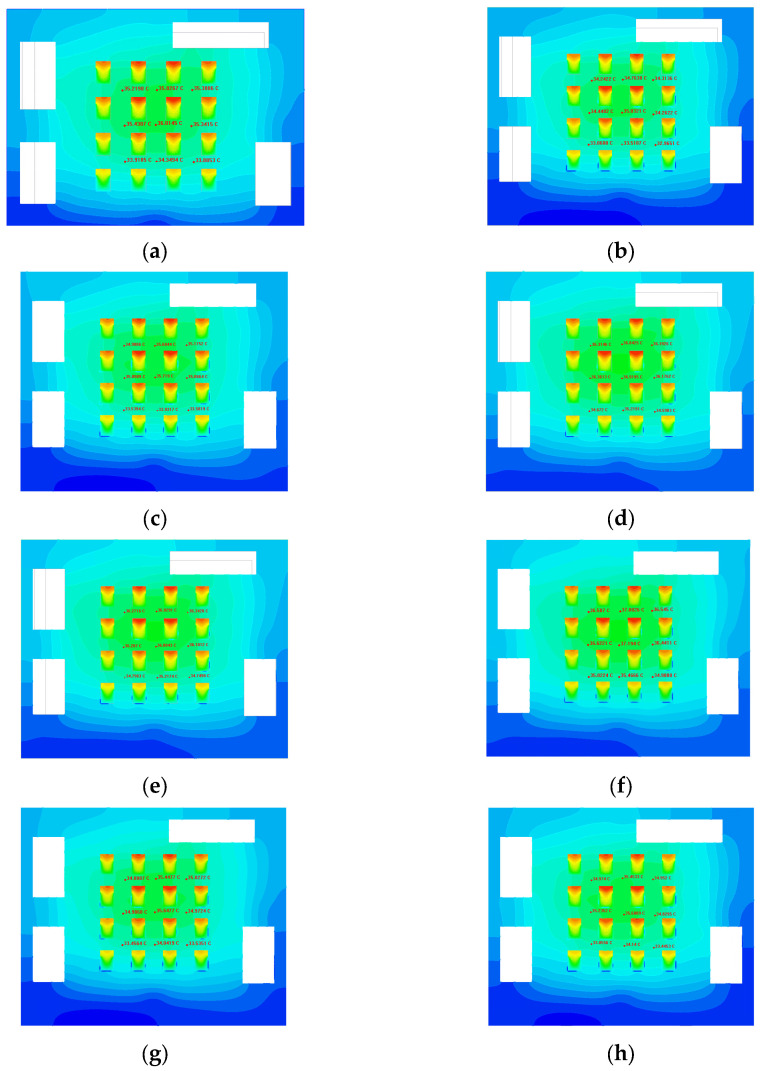
Temperature of the monitoring point on the surface of the heat source carrying plate, and the red areas indicate hot spots: (**a**) topology optimization I channel; (**b**) series channel; (**c**) circular pin rib channel; (**d**) parallel I channel; (**e**) parallel II channel; (**f**) triangular pin rib channel; (**g**) tree channel; (**h**) topology optimization II channel.

In order to correspond to the experiments, we selected the same nine temperature monitoring points on the heat source surface carrying plate that is able to be monitored by the experiments. We extracted the monitoring point temperatures of different liquid-cooled heat sink structures, as shown in Figure 12. The impact of different heat sinks on temperature distribution can be reflected by analyzing the monitoring point temperatures.

**Figure 13:**
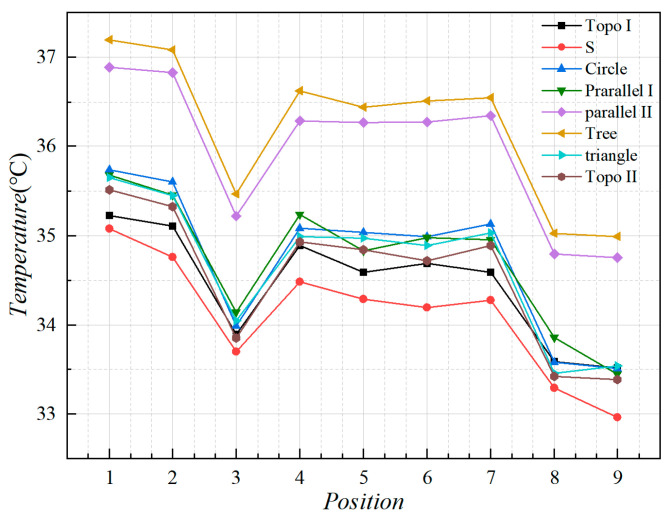
The junction temperature of each heat source in steady-state liquid-cooled environment.

We plotted the temperatures at the monitoring points on the surface of the carrying plate as shown in Figure 13. Analysis of the temperature monitoring point data revealed that the temperature rise in the series channel is the lowest. The temperature of the topology optimization channels I and II, circular and triangular pin ribbed channels, and the parallel I channel were close to each other and had lower temperatures. The tree and parallel II channel had the highest temperature.

#### 3.3.2. Simulation Results of Fluid Characteristics

**Figure 14:**
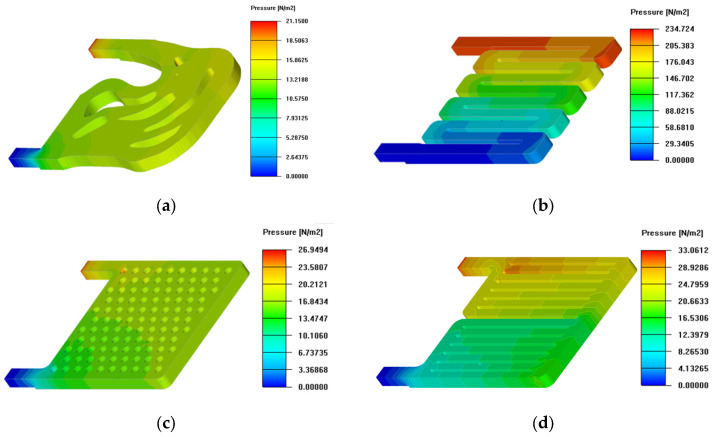

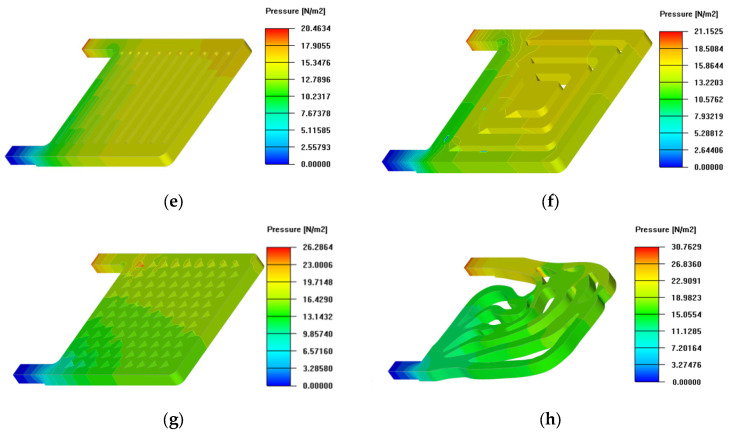
Pressure contour of liquid-cooled channels: (**a**) topology optimization I channel; (**b**) series channel; (**c**) circular pin rib channel; (**d**) parallel I channel; (**e**) parallel II channel; (**f**) triangular pin rib channel; (**g**) tree channel; (**h**) topology optimization II channel.

To analyze the fluid characteristics in the channel and the pressure loss of the liquid-cooled, we obtained the pressure distribution of the fluid through numerical simulation. The pressure drop contour is shown in Figure 14, which clearly demonstrates the trend of pressure change in the channel. In particular, the pressure gradient increases distinctly at the channel’s corners, narrow regions, and branch points, indicating that these areas are the main contributors to pressure drop.

**Figure 15:**
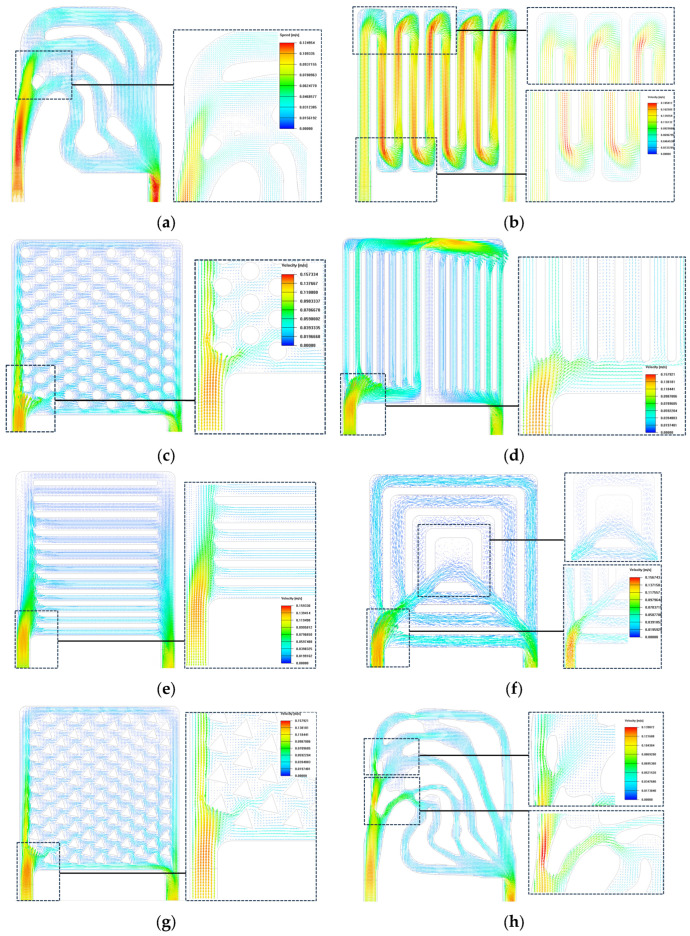
Velocity vector contour of liquid-cooled channels: (**a**) topology optimization I channel; (**b**) series channel; (**c**) circular pin rib channel; (**d**) parallel I channel; (**e**) parallel II channel; (**f**) triangular pin rib channel; (**g**) tree channel; (**h**) topology optimization II channel.

In order to analyze the fluid characteristics of each heat sink, the fluid state of the coolant in the liquid-cooled device was simulated by the simulation software, and the flow direction and flow rate of the coolant were presented by vector contour diagrams, as represented in contour Figure 15. In Figure 15d–f, the flow velocity contour diagram shows that the coolant becomes slower when the fluid passes through the secondary flow channel. The reason for this nonuniformity in pressure and velocity is as follows: as the coolant fluid is distributed across the channels, the volumetric flow rate decreases. Thus, following Bernoulli’s principle, the hydrostatic pressure grows in the direction of flow. Nevertheless, the pressure declines along the flow path because of the frictional force from the primary walls, which gives rise to poor flow distribution.

Figure 15b shows the flow velocity contour of the series channel; the flow velocity distribution is more irregular, and the corners of the flow channel cause larger pressure loss and a vortex. As in Figure 15c,g, the flow velocity distributions of the circular pin ribs and the triangular pin ribs are similar, with a gradual decrease in the fluid away from the entrance and exit. Vortices are generated near the localized ribs of the triangular needle rib.

Conversely, in the topology optimization, the pressure drop across the flow channel shows relative uniformity, as illustrated in Figure 14a,h. Figure 15a,h shows that a preferable flow distribution is observed in the topo design. The tendency can be explained in the following way: the flow goes through a converging section (cross-section decreasing), which is indicated by double-sided arrows in Figure 15a. Consequently, the upward trend in flow rate, in turn, results in lower static pressure. Wall friction also tends to reduce hydrostatic pressure, thereby contributing to a more even flow distribution. Additionally, the rounded corners—emphasized with dotted arrows in Figure 15a,h—prevent the occurrence of vortices at these corners. Moreover, the branch-shaped channel arrangement can lower pressure drop, a result of the minimum power dissipation term in the multi-objective function as stated in Equation (6).

To contrast the fluid properties of various channel constructions more intuitively, the pressure values Pin, Pout of the liquid-cooled model are obtained, and the pressure drop ΔP of the runner structure is obtained from the disparity between the inlet pressure and outlet pressure, as outlined in Equation (12).

(12) $$\Delta P=P_{\mathrm{in}}-P_{\mathrm{out}}$$

**Figure 16:**
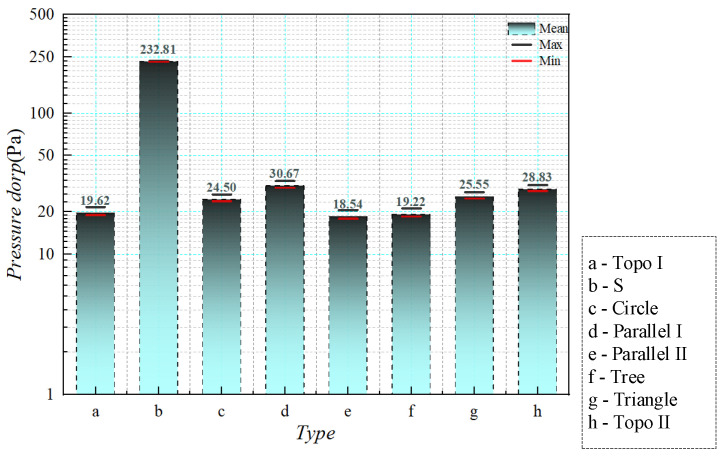
Numerical simulation of pressure drop with flow rate of 0.1 m/s.

Combined with the numerical calculation of pressure drop in the finite element numerical simulation, the calculation results are shown in Figure 16, in which the S channel’s pressure fall is the greatest; it surpasses that of other flow channels by a significant amount. In contrast, the channel structures with the smallest pressure drop are the topology optimization I, tree, and parallel II, respectively. The main characteristics of these channels are that the inlet and outlet are relatively close to each other and the branch widths of the runners are relatively wide, so that the fluid energy dissipation is relatively reduced. For circular and triangular pin ribs, the channel structures are similar, but the circular ribs are relatively favorable for fluid flow, resulting in a slightly lower pressure drop than the triangular pin ribs. In addition to the S channel, the pressure drop is relatively high for the parallel I and topology optimization II channels, both of which have more branches and are more elongated. The parallel I has a longer flow at the inlet and outlet, leading to a higher pressure drop. The topology optimization II has only 0.5 fluid percentage in the runner structure, which is also the reason for its thin branches, and the pressure fall significantly declines after increasing the fluid percentage to 0.7.

The above analysis may be summarized as follows:

The adaptive flow channels generated by topology optimization I closely follow natural flow paths, resulting in low flow resistance (as evidenced by the gentle color gradient in the pressure drop contour plot and low pressure drop values). Heat is dissipated uniformly and efficiently, resulting in moderate-to-low heat source temperatures with minimal fluctuations. Low resistance ensures adequate coolant flow, while uniform velocity distribution maintains consistent convective heat transfer coefficients across all heat source regions. This uniform and efficient heat removal contributes to moderate-to-low heat source temperatures with minimal variation.

Series channel: The extended flow path accumulates friction losses, evident in the steep color gradient of the pressure drop contour plot (the highest pressure drop value among all channels). Flow velocity gradually diminishes along the flow direction, with high velocity at the inlet section and low velocity at the rear end, featuring minor localized flow dead zones. High resistance results in relatively high inlet velocity, enabling strong convective heat transfer and low temperatures near the inlet heat source. However, coolant temperature gradually increases from inlet to outlet, causing weakened heat transfer at the rear heat source due to elevated coolant temperature and slightly higher temperatures there. Overall, this presents a ‘low-front, high-rear’ yet moderately low temperature distribution.

Circular pin rib flow channel: The pin ribs increase flow resistance, with the pressure drop contour map exhibiting a moderate color gradient (indicating a moderate pressure drop value). Significant flow disturbance occurs around the pin ribs (resulting in localized high flow velocities), yet micro-dead zones readily form within the pin gaps. The ‘disturbance effect’ of the pin fins enhances local convective heat transfer coefficients. However, the low heat transfer coefficient in dead zones contrasts with the high heat transfer in high-velocity zones, resulting in moderate heat source temperatures with fluctuating distributions.

The Parallel I channel features a multi-branch parallel structure. Flow splitting balances branch resistance, yielding a gentle color gradient in the pressure drop contour map (indicating a moderately low total pressure drop). Parallel flow distribution ensures adequate flow rates across all heat source regions, with fin-rib disturbance further enhancing heat transfer. However, minute resistance differences between branches cause a slight flow distribution imbalance, resulting in moderately fluctuating heat source temperatures.

Parallel II channels feature a multi-branch parallel structure with complex geometry. Significant resistance variations between branches produce uneven color gradients in the pressure drop contour map (total pressure drop is moderately high). Flow velocity distribution across parallel branches is uneven, with some branches exhibiting markedly insufficient flow (sparse regions visible in velocity vector plots). Uneven branch flow significantly reduces convective heat transfer coefficients, preventing timely heat removal. Consequently, heat source temperatures are generally elevated with substantial fluctuations.

Triangular pin rib channels employ triangular pin fins, offering lower flow resistance than circular pin fins. Pressure drop contour plots exhibit a smoother color gradient (resulting in lower total pressure drop). Flow velocity distribution is more uniform, with triangular pin fins inducing relatively gentle turbulence and better flow field stability. Low resistance ensures adequate flow rates, while the moderate turbulence from triangular pin fins enhances heat transfer without causing excessive flow field irregularities. Consequently, heat source temperatures are lower and exhibit a smoother distribution.

Tree channels exhibit a hierarchical structure from the main trunk to branches, with a corresponding hierarchical distribution of resistance. The color gradient in the pressure drop contour plot is moderate (indicating moderate total pressure drop). Flow gradually diverges from the main trunk towards terminal branches, where velocity significantly attenuates (evidenced by dim colors and sparse velocity vectors in the terminal regions). The low flow velocity at branch terminations causes a substantial reduction in convective heat transfer coefficients, leading to heat accumulation at corresponding heat sources (particularly distant chips). Consequently, heat source temperatures are generally elevated with significant fluctuations.

Topology optimization II channels are customized flow paths generated through topology optimization, highly matched to the multi-heat-source layout. The pressure drop contour map exhibits a gentle color gradient (moderate resistance). Flow velocity distribution is continuous with excellent uniformity, with channels perfectly aligned to the required heat flow paths. The adaptive channel geometry to multiple heat sources ensures sufficient and uniform flow velocities across all regions, enabling efficient convective heat transfer with minimal variation. Consequently, heat source temperatures are minimized with the most stable distribution. Compared to Topology optimization I, this channel exhibits a volume ratio reduction of less than 20% while delivering superior cooling performance. The increased designable area not only enhances heat exchange between fluid and walls but also provides additional optimization gains for the channel configuration.

## 4. Experimental Verification

In order to further verify the heat dissipation performance and fluid characteristics of the liquid-cooled heat sink at the same side inlet and outlet, based on the liquid-cooled design obtained by the above topology optimization, a variety of liquid-cooled heat sinks were manufactured by CNC. And a experimental platform of liquid-cooled heat sinks was built to characterize the heat dissipation performance and fluid characteristics of different liquid-cooled heat sinks.

### 4.1. Experimental Setup

The original temperature of the liquid coolant and the environment temperature was 25 °C, and the original atmospheric pressure was 101 kPa. The heat dissipation performance and fluid characteristics of the liquid-cooled heat sink were investigated, and the heat sink samples were designed and manufactured. In addition, a complete liquid-cooled experimental platform was designed and built to accurately observe the pressure fall at the inlet and outlet and the temperature distribution over the tool surface under different fluid flows.

#### 4.1.1. Experimental Platform

**Figure 17:**
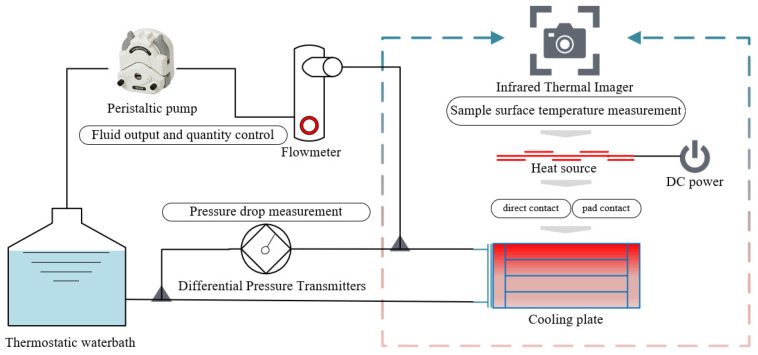
Schematic diagram of liquid-cooled experimental platform.

The core components of the liquid-cooled experimental platform include a liquid-cooled tank, peristaltic pump, flow meter, differential pressure transmitter, infrared thermal imager, and DC power supply, all of which work together to provide reliable experimental data support. The operating principle of the liquid-cooled experimental platform is shown in Figure 17. Liquid-cooled tank provides liquid coolant at steady temperature; The liquid coolant was extracted by the peristaltic pump from the liquid-cooled tank and input to the heat sink; the flow meter controlled the inlet speed of the liquid coolant of the experiment (with a measuring range of 20 mL/min–160 mL/min) to obtain the target parameters under a certain flow rate under different flow channel types. Infrared thermal imaging was utilized to detect the temperature of the tool on the heat source surface. The differential pressure transmitter was used to calculate the disparity between the outlet and inlet of the liquid-cooled (the experimental test differential pressure included the loss of pressure drop along the hose transporting the liquid coolant).

**Figure 18:**
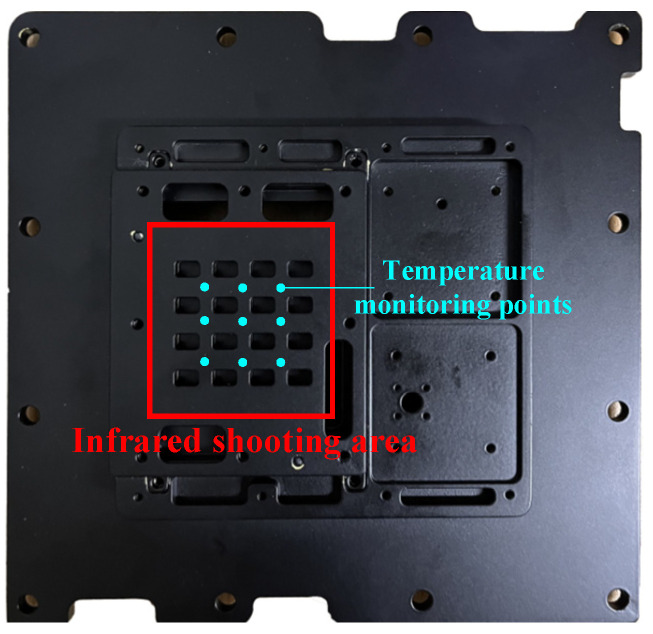
Infrared thermal imager shooting area.

Consistent with the simulation model, the T/R component was the source of high heat flux, and sixteen MCHs were used to simulate the T/R components. We fixed the MCH to the heat sink using a carrying plate, and used a thermal pad to fill the gap between the MCH and the heat sink. Within the liquid-cooled experimental platform, the shooting area of the infrared thermal imager was the surface where the heat source was located, and nine temperature monitoring points were provided for comparing the heat dissipation characteristics of various path structures, with details illustrated in Figure 18.

**Figure 19:**
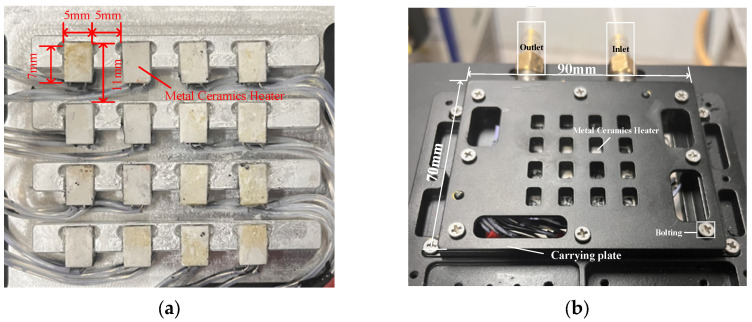
(**a**) Experimental structure of heat source assembly; (**b**) experimental structure of liquid-cooled heat sink after assembly.

The equipment of the liquid-cooled experimental platform was connected through the hose conveying the liquid coolant. Sixteen MCH were fixed on the PCB, and the heat source was connected to the DC power supply in parallel through the wire. After the heat source was installed, as presented in Figure 19a, the MCH had a size of 7 mm × 5 mm and a thickness of 1.5 mm. The heat conduction pad was set between the thermal source and thermal sink. Assembling the thermal conductive pad helped avoid thermal resistance between the heat source and heat sink, which aligned with actual working conditions. After assembly of the above parts, it is shown in Figure 19b. The whole design fully considers the test accuracy and the convenience of actual operation, and provides an important experimental basis for further optimization.

The inlet and outlet of the liquid-cooled sink were connected to the hose through the flange, and the liquid coolant in the liquid-cooled tank was circulated through the hose. The sealing of the liquid-cooled system is very important, and the leakage of the liquid coolant will not only lead to the deviation of the fluid characteristics measurement, but also lead to the short circuit faults of the heat source in the water.

#### 4.1.2. Design and Manufacture of Samples

**Figure 20:**
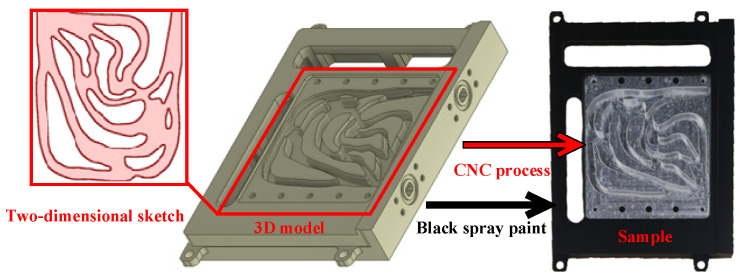
Three-dimensional model of liquid-cooled heat sink equipped with heat source.

The above topology optimization results and designed flow channels are two-dimensional drawings. According to the designable area, a three-dimensional model of the above two-dimensional structure was established by stretching. As illustrated in Figure 20, the outcomes of the topology optimization were converted into CAD sketches, and the sketches were brought into the designed support structure for 3D modeling. Finally, the aluminum alloy sample was machined using CNC, and the sample’s surface was sprayed with matte black paint. Then the surface of the flow channel was sealed by a cover plate during the subsequent experiment.

**Figure 21:**
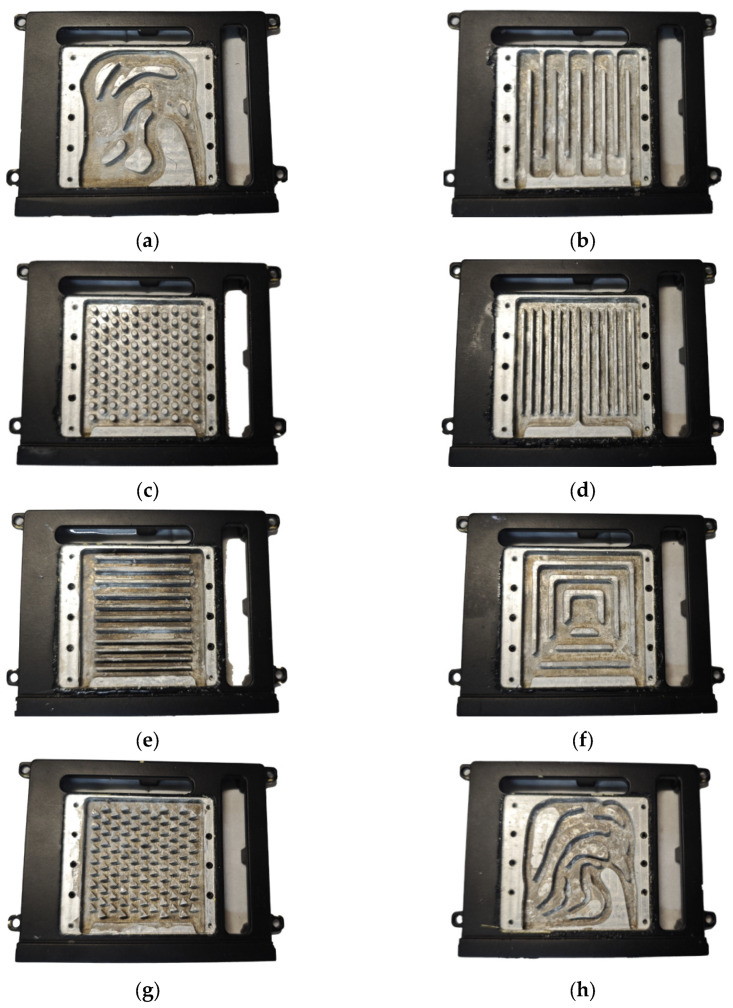
Eight channel designs in heat sinks for experiment: (**a**) topology optimization I channel; (**b**) series channel; (**c**) parallel I channel; (**d**) circular pin rib channel; (**e**) triangular pin rib channel; (**f**) parallel II channel; (**g**) tree channel; (**h**) topology optimization II channel.

Based on the manufacturing process illustrated in Figure 20, eight types of liquid-cooled channel heat sinks were manufactured as shown in Figure 21.

#### 4.1.3. Obtaining of Experimental Results

**Figure 22:**
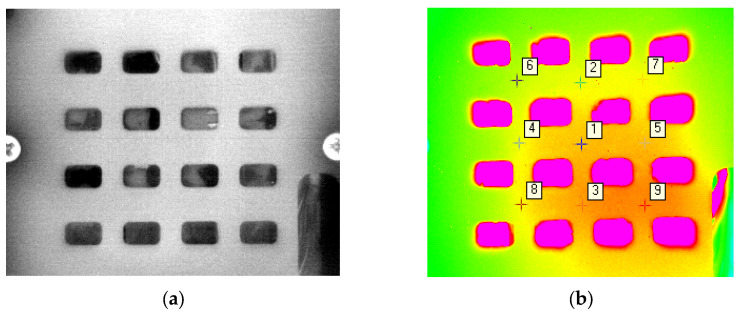
(**a**) Surface of the heat source photographed by the infrared thermal imager after focal shooting area; (**b**) surface monitoring points (position of the ‘+’) through infrared thermal imaging.

The heat dispersion performance of different liquid-cooled sinks was studied through the experiment. Sixteen MCHs were interconnected to DC power through wires. DC power provided 25.6 W of heat source, and each MCH in the circuit was 1.6 W of power, simulating the heat of four 0.4 W chips in a four-channel T/R component. And the thermal conductive pad was mounted between the liquid-cooled sink and the heat source. Since the area photographed by the infrared thermal imager needs to be on a plane, the heat source situated on the carrying plate’s surface cannot be directly obtained by the thermal imager. Nine temperature monitoring points on the surface of the carrying plate were recorded, and the monitoring conditions of the heat source surface and surface monitoring points through infrared thermal imaging are shown in Figure 22.

Through obtaining the temperature of monitoring points, the heat dissipation effectiveness of different liquid-cooled sinks can be assessed using an indirect method. The uniformity of heat source temperature is also very important, so we calculate the standard deviation to compare the influence of the liquid-cooled heat sink on heat source temperature uniformity.

In the liquid-cooled experiment, pressure drop is the main characteristic that defines the fluid behavior of the liquid-cooled thermal sink, which represents the pressure loss during the process of fluid inlet to outlet. The pressure difference transmitter obtains the pressure at the inlet and outlet of the liquid-cooled heat sink, named Pin and Pout, respectively. The liquid-cooled fluid inlet of the liquid-cooled sink controls the flow through the flow meter. The flow range was 20 mL/min–160 mL/min, and the flow channel inlet was controlled by adjusting the flow meter knob. The data were observed every 10 mL/min, and the flow channel pressure drop data were recorded through the pressure difference transmitter.

### 4.2. Experimental Results and Discussion

Through the analysis of empirical data, this research further discusses the heat and fluid features of various channels in heat sinks, including topology optimization channels, and compares them with the simulation results.

#### 4.2.1. Temperature Results

**Figure 23:**
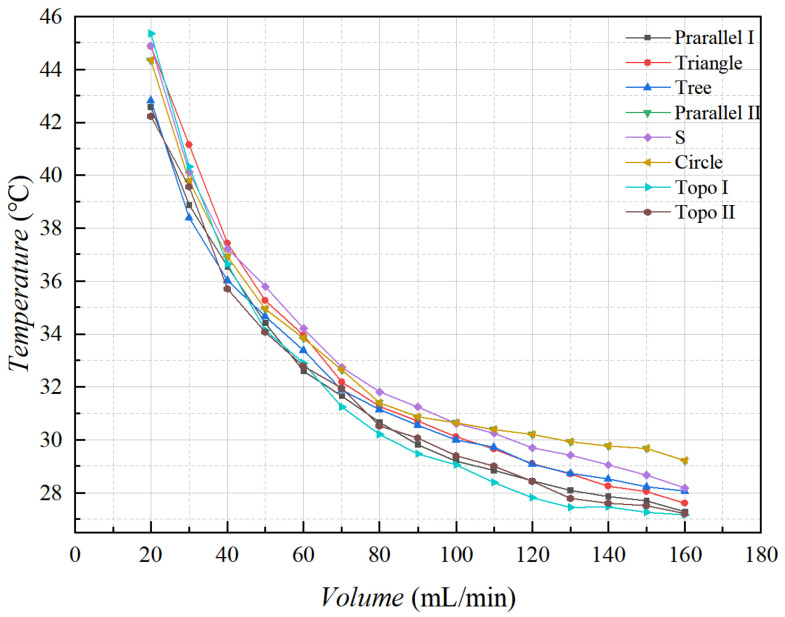
The average temperature of the monitoring point of the carrying plate with different flow rates.

By observing the surface of the carrying plate under steady state in the liquid-cooled experiment, nine monitoring points on the surface of the carrying plate were observed, as illustrated in Figure 16. The average temperature of the monitoring point on the surface of the carrying plate was obtained, as shown in Figure 23.

The results indicate that the average temperature of topology optimization I, II, and parallel I channel heat sinks was relatively low when the flow rate exceeded 80 mL/min. The average temperature of series and circular pin rib channel heat sinks was relatively high when the flow rate exceeded 80 mL/min, and the overall temperature difference in other channel types was small. We selected flow rates below 50 mL/min for comparison, because when the flow rate is below 80 mL/min, the maximum temperature distribution can not show a clear pattern. Following the above analysis, the heat sources of topology optimization II heat sink with the average temperatures decreased by 4% relative to other flow channels, and topology optimization I heat sink decreased by 6%.

**Figure 24:**
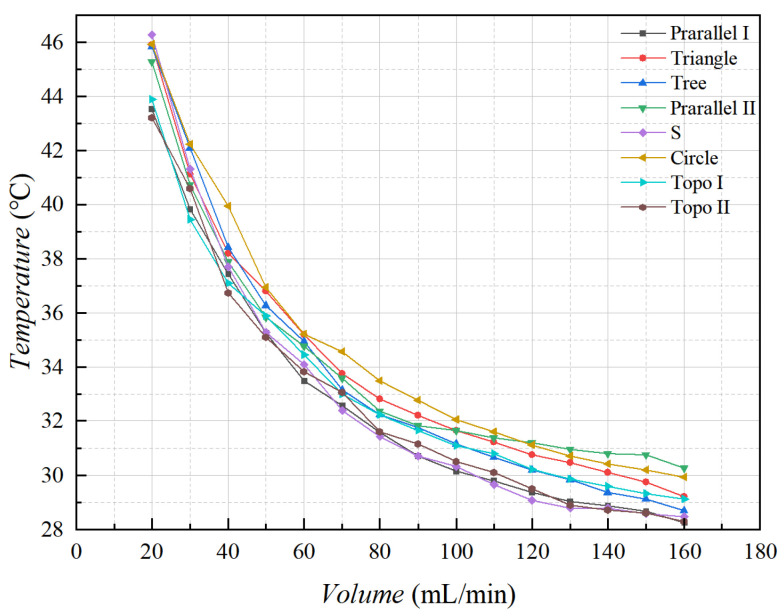
Variation in the maximum temperature at the monitoring point of the carrying plate with different flow rates.

From the overall temperature situation (shown in Figure 24), it was evident that the maximum temperature of the heat sources with the series, topology optimization II, and parallel I channel heat sinks was relatively low when the flow rate exceeded 80 mL/min. The maximum temperature of the heat sources with parallel II channel and circular pin rib channel was relatively high when the flow rate exceeded 80 mL/min. The rest of the heat sinks were in the intermediate level. Following the above analysis, the heat sources of topology optimization II channel with the maximum temperatures decreased by 8% relative to other flow channels, and topology optimization I decreased by 4%.

**Figure 25:**
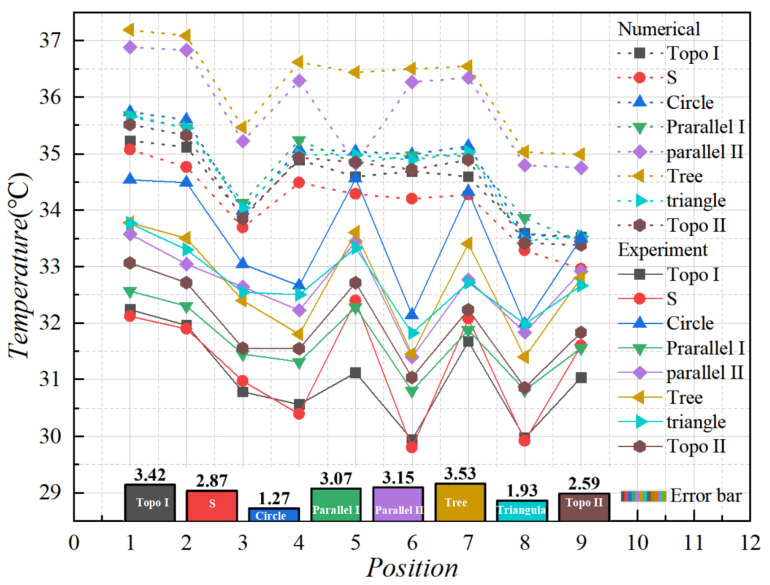
Temperature results from numerical simulations and experiments at monitoring points.

The flow velocity of 80 mL/min was selected and compared with that in the numerical simulation in Section 3. As shown in Figure 25, the simulated temperatures were mildly higher than those in the experimental test, and the overall temperature distribution trend of different liquid-cooled runner structures was basically the same. Following the calculation, the temperature error between the test and simulation did not exceed 10%.

**Figure 26:**
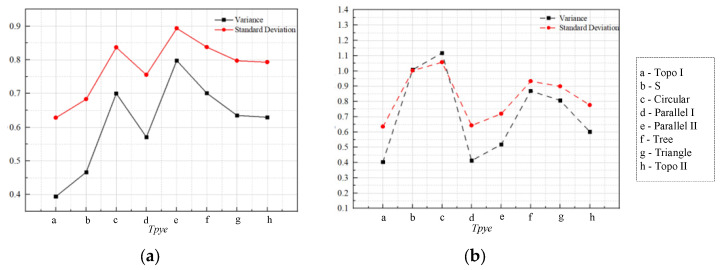
(**a**) Variance of monitoring point in numerical simulation and experiments; (**b**) standard deviation of monitoring point in numerical simulation and experiments.

In the temperature of the liquid-cooled experiment, the standard deviation and variance between the monitoring points of the heat source carrying plate’s surface temperature are shown in Figure 26. It can be found that the variance and standard deviation of topology optimization I channel, series channel, and parallel I channel were small; the variance and standard deviation of circular and triangular pin rib channel, parallel II channel, and tree channel were large; and topology optimization II was at the middle level.

According to the above comparative analysis of numerical simulation and experimental results, it was evident that the topology optimization I channel and the S channel had good heat dissipation performance. The simulation outcomes aligned with the test data in the overall trend, but there was a certain deviation in the specific values; the deviation range was about 10% or less. The reason for this deviation is that the finite element simulation results are affected by the mesh, and the laminar flow model does not consider the turbulent flow. However, during the experimental test, some turbulence may exist in the liquid-cooled flow. At the same time, the power supply wire and actual output power influence the MCH’s heat dissipation, resulting in a certain deviation.

#### 4.2.2. Pressure Drop Results

**Figure 27:**
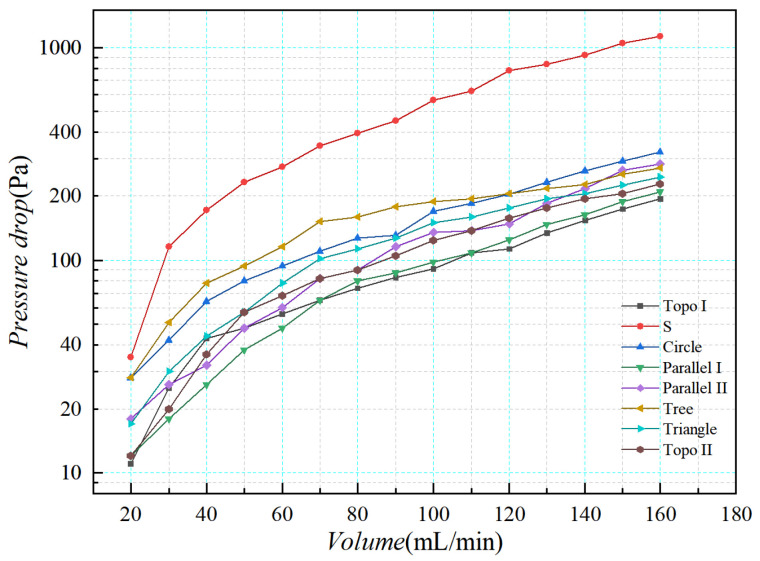
Inlet and outlet pressure drop curves of different channel structures with various flow rates.

The flow resistance data of the liquid-cooled experiment test were obtained through the pressure difference transmitter in the experimental platform. In order to compare the flow resistance of eight kinds of liquid-cooled sinks in the liquid-cooled experiment, the pressure difference data of each inlet and outlet under steady flow were observed, as shown in Figure 27. According to the data, the pressure drop in the series channel was distinctly higher compared to other flow channels. Topology optimization I channel and parallel II channel had the lowest voltage drop. Under the flow rate of 20 mL/min–50 mL/min, the pressure fall trend of these eight flow channels gradually decreased. When the flow rate reached 50 mL/min, the rising trend of pressure drop in each flow channel gradually stabilized as the flow rate continued to increase. Meanwhile, the pressure drop of the series channel rose steadily with the increasing flow rate, while the pressure drop difference between the other flow channels narrowed gradually as the flow rate went up. Based on the above analysis, topology optimization I channel exhibited the most favorable fluid characteristics, with a pressure drop 9% lower than that of the parallel flow channel and significantly superior to the other channels. Topology optimization II channel also demonstrated commendable fluid properties, alongside increased flow rates, and achieved a more advantageous pressure drop compared to the other channels.

In general, a higher flow rate corresponds to greater convective heat transfer, and therefore, more heat can be dissipated from the heat source, thus maintaining the temperature distribution to be more consistent. The flow resistance data obtained in the above liquid-cooled experiment is the combined pressure drop of the liquid-cooled heat sink’s flow path and the infusion hose. Therefore, the fluid pressure drop in the experiment is able to be obtained by calculating the flow resistance consumed by the fluid in the hose in the experiment and accounting for the discrepancy between the pressure drop obtained in the experiment and the flow resistance along the process. The flow resistance calculation formula along the process is shown in Equation (13).

(13) $$\Delta P_{l}=f\frac{l}{D_{h}}\frac{\rho v^{2}}{2g}$$

l is the length of the flow channel; v is fluid velocity, and its calculation formula is shown in (14). A represents the cross-sectional area in the direction of fluid flow, and u represents the flow rate; ρ refers to the fluid density; Dh is equivalent diameter; f is the resistance coefficient along the path. When f is laminar flow (Re ≤ 2320), its calculation formula is shown in (15). The Reynolds number Re is calculated by the formula shown in Equation (16).

(14) v=uA

(15) f=64Re

(16) Re=ρvdμ

**Figure 28:**
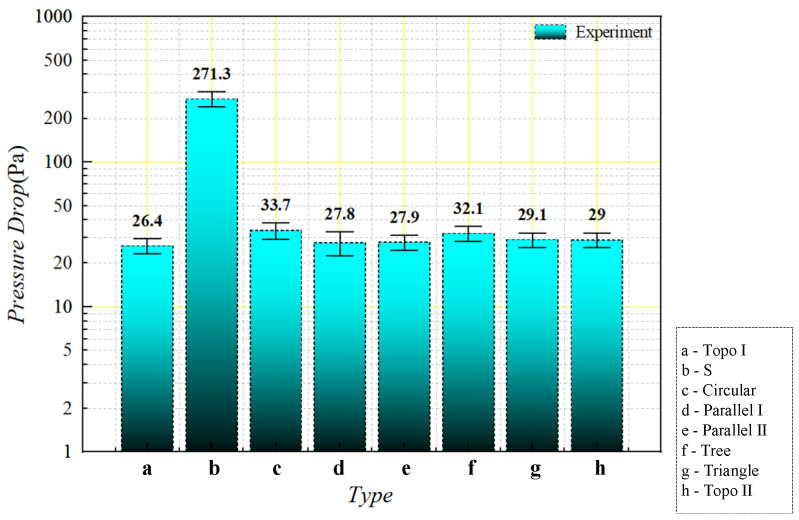
Comparison of experimental pressure drop values of liquid-cooled experiment with different flow channel types at inlet flow rate of 0.1 m/s.

The actual pressure drop corresponding to the flow path structure was acquired by calculating the flow resistance along the flow path of 80 mL/min (the inlet flow rate of the liquid-cooled heat sink is about 0.1 m/s) according to the above formula (shown in Figure 28). The experimental data was due to the possibility of sample processing errors in the experiment, and a few impurities in the deionized water after several experiments. There was a slight error in the experimental results.

A comprehensive analysis of the heat dissipation performance and fluid characteristics of various flow channels was carried out. The topology-optimized I, II, and parallel I channels showed good performance regarding thermal transmission efficiency and pressure drop characteristics. The series channel had excellent thermal management capability, but the much higher pressure drops than the other flow paths may adversely affect the reliability of the liquid-cooled heat sink. The tree and parallel II channels were less efficient due to the uneven distribution of flow rates in each channel branch. The circular and triangular pin rib channels exhibited similar heat dissipation and fluid performance to the parallel II channel, with uneven flow distribution resulting in average heat sink capacity.

**Figure 29:**
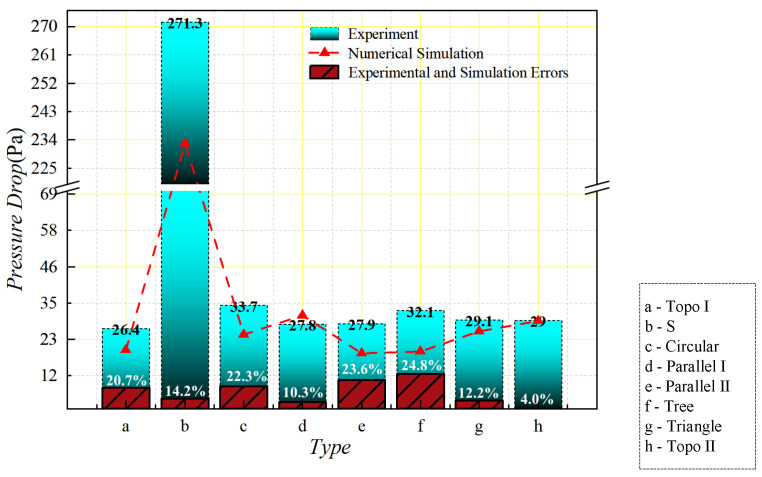
Comparison of numerical simulation and experimental pressure drop values of different flow channels at 0.1 m/s flow rate.

After comparing the pressure drop results from the tests and numerical simulations, the findings are shown in Figure 29. From the figure, the experimental data are slightly higher than the simulation data, but the deviation of each heatsink is acceptable. The reason for this is that there may have been sample processing errors in the experiment, and the deionized water may have had a little impurity after many experiments; the actual experimental pressure drop was slightly higher than the simulation data.

## 5. Conclusions

In this study, we designed various flow channels for liquid-cooled heat sinks and obtained flow channels targeting temperature uniformity and fluid dissipation through topology optimization. The heat and fluid features of the liquid-cooled sink were investigated through numerical simulation. The results demonstrated that the topology optimization channels exhibit superior performance in both thermal and fluid characteristics, particularly in heat source temperature uniformity and pressure drop reduction, outperforming traditional flow channels. The topology optimization I and II channels achieved an optimal balance between thermal dissipation and fluid performance, effectively lowering heat source temperatures while minimizing pressure drop losses.

Furthermore, we evaluated the liquid-cooled heat sinks: topology optimization I, series, parallel I, circular pin rib, triangular pin rib, parallel II, tree, and topology optimization II channel through experiment. Consistent with the results of numerical simulation, the experimental data revealed that the topology optimization I, II, and parallel type I flow channels delivered the best thermal performance, maintaining low and uniform heat source temperatures. While the series channel demonstrated good heat dissipation capability, its significantly higher pressure drop made it unsuitable for long-term stable operation in practical applications. The pin rib flow channels (both circular and triangular) showed comparable thermal and fluid characteristics but exhibited poorer temperature uniformity. Due to their design characteristics, both the tree and parallel II channels exhibited inferior thermal performance despite their low pressure drop. The topology optimization II channel offered an excellent compromise between thermal dissipation and fluid characteristics, making it particularly suitable for high heat flux environments. Although the series channel provided effective cooling, its inherent high pressure drop limited its applicability. Moreover, through the simulation and experimentation, we obtained the following quantitative results: The average temperatures of the heat sources in topology optimization I and II channel were 6% and 4% lower than those in the other flow channels, respectively, and topology optimization I channel exhibited the most favourable fluid characteristics, with an pressure drop 9% lower than that of the parallel flow channel.

Through topology optimization and experimental validation, this study successfully developed an optimized liquid-cooled heat sink for a millimeter-wave antenna. The results confirm that the topology optimization design provides significant advantages in both thermal performance and fluid characteristics, effectively reducing heat source temperatures while minimizing fluid flow pressure losses. These findings offer novel design concepts and methodologies for heat management of high-power electronic devices, demonstrating substantial engineering application value.

## Figures and Tables

**Table 1:** The parameters of the topology optimization.

q	Da	V_0_	L (mm)	γ_β_	μ (N·s/m^2^)
0.01	1 × 10^−4^	0.5	4	0.5	0.001

**Table 2:** Material parameters of liquid-cooled heat sink.

Component	Material	Density ρ (kg/m^3^)	Heat Conductivity k (W(m·K))	Specific Heat (J/kg K) Cp	Dynamic Viscosity μ (N·s/m^2^)
Heat sink	Aluminum 6061	2700	240	336	—
Carrying plate	Aluminum 6061	2700	240	336	—
MCH	96 Alumina	3970	15	61	—
Thermal pad	Alumina	3200	3	1000	—
Coolant	Deionized water	998.2	0.61	4180	0.001

**Table 3:** Validation of mesh independence.

Mesh Number	$T_{\mathrm{topo}I-1,\max}$ [°C]	$|(T_{\mathrm{topo}I-1,\max}^{m}-T_{\mathrm{topo}I-1,\max}^{m+1})/T_{\mathrm{topo}I-1,\max}^{m}|$
968,718	51.5	--
1,736,644	48.8	5.5%
2,902,755	47	3.8%
4,308,285	45.9	2.4%
6,568,243	45.6	0.7%

## Data Availability

The original contributions presented in this study are included in the article. Further inquiries can be directed to the corresponding author.
